# Journey through the Trials and Triumphs of Disability

**DOI:** 10.4102/ajod.v1i1.16

**Published:** 2012-09-28

**Authors:** Helen L. Laas

**Affiliations:** 1 South African Association for Learning and Educational Differences, KwaZulu-Natal branch, South Africa; 2 Inclusive Education, Embury Institute for Teacher Education, Durban, South Africa

## Abstract

***One Woman’s journey through the Trials and Triumphs of Disability***, **Disabled Peoples International 8th World Assembly 2011 Durban, South Africa, October 10–13, 2011.** When embarking on my career as a teacher at a special school in South Africa, I never thought that a motor vehicle accident would place me in the position where my learners with disabilities suddenly saw me as an ally. Little did I realise the chasm that exists between able-bodied people and people with disabilities, or the remarkable role I would find myself in whilst actively addressing disability and Inclusive Education issues. My experiences with disability in South Africa drew encouraging attention from delegates at the Disabled People’s International 8^th^ World Assembly when I shared my story. The resounding positive response affirmed that my experiences are not unique to nationality, gender, race or age, and are typical of the time and country in which I live, where people with disabilities are considered to have little potential, and woman with disabilities are further marginalised. In the infancy of our democracy, we are still in the early days of attending to equity amongst all South Africans. This story comprises both a narrative and a graphic presentation which run parallel, although not always telling an identical story; they complement one another and should be experienced simultaneously. Ultimately, it relates the success that can be achieved by pro-active people with disabilities as members of the South African society within their own spheres of knowledge and skill to change attitudes and practices of people without disabilities in education and local communities.

## Introduction

### My story

My story starts off with a glibly-brief summary of the injuries that I sustained that should suffice for those whose focus still lies within the medical model. Everybody’s story begins within themselves. Mine began within an average body and a gifted mind. Today, I have a less than fully functioning body, nerve damage, loss of sensation, varying degrees of limitations in mobility and function, permanent pain, depression, post-traumatic stress disorder, and some loss of intellectual functioning although I am still classified as being on the gifted end of the scale. On this level, I have grieved and accepted my loss and redefined my identity. But if that is all there was to it, there would be nothing more to this story and there *is* much more to my story.

It is only when we move out of the medical model and into the social realm that we really realise the repercussions of one moment.

Life as a woman with a disability is not easy, but then again, nobody said life was ever going to be easy.

My experience is that the combination of misunderstanding of disability and gender bias creates a chasm in interaction with the ’normal‘ populace. As a lone individual, I have found that it is necessary to actively champion the breaking down of illusions that people carry before we truly can begin to make real changes. Eliminating discrimination at every level forms a core part of not only the international disability movement, but also of my own personal purpose. Tied in with this cause is advocating the act of ownership that our disabilities are only one part of ourselves and not our defining factor amongst youth with disabilities.

Every person traverses their own life. Looking back, the major events and choices form crossroads that clearly delineate each segment of my journey from the last, forming chapters of my life thus far. This is my story.

#### Significance of the study

This study outlines a teacher’s experiences in disability and Inclusive Education from viewpoints both before and after facing disability on a personal level. It exposes the complexities of the changing of identities that one can undergo in one’s life as a result of disability, as well as evaluating the effectiveness of Inclusive Education training on undergraduate education students and teachers in both urban and rural settings, and examining possible ways forward. This story comprises both a narrative and a graphic presentation which run parallel, although not always telling an identical story; they complement one another and should be experienced simultaneously.

## Research methods and design

This qualitative study draws on narrative reflections by the author as main participant, and combines the views of ex-learners and students taught by the author to create a story and visual presentation of her experiences (Henning 2004). The author’s experiences of disability and the effects thereof are viewed in both the medical and social models (Landsberg 2004). Within the social model, an ecosystemic model is utilised to view the experiences of the author, specifically focussing on the microsystems and chronosystems which consider the systems directly interacting with the author over a period of time set within the South African context (exosystem and macrosystem) (Donald, Lazarus & Lolwana 2010, Landsberg 2004).

Falling into the category of narratives Hiles, Čermák and Chrz (2009) describe as ‘dominat[ing] human discourse, offering a major resource for providing accounts of events, as well as the social and cultural practices for the circulation of meanings’, this study constructs the author’s experiences and views in South Africa from the period preceding a motor vehicle accident in 2007, to 2011, forming four distinct chapters, documented as both a narrative and a visual presentation.

In the process of reflecting upon effects of decisions made by the author, further research was carried out to determine the validity of the author’s impressions. Two smaller studies (hereafter referred to as Study A and Study B), utilising purposive sampling, were thus included within the main reflection to ascertain the effect that the author’s disability has had on those within the microsystem interacting with her, specifically within the sphere of education. Studies A and B employed both closed and open-ended questions which combined to give a rich view of the participant’s impressions. Study A consisted of learners with a variety of disabilities who completed Grade 12 in 2010. Participants had been taught choir in 2006–2007 before the accident and Computer Applications Technology in 2009 and 2010 by the author. They were therefore able to give a comparative assessment of her as a teacher both pre-accident and post-accident, as well as draw a comparison with able-bodied teachers. Study B consisted of students attending a private tertiary institution which specialises in teacher education, who were lectured by the author on Inclusive Education and were asked to evaluate the impact the author’s disability had on their views of people with disabilities as well as inclusive teaching practices.

## Results

The narrative and graphic reflections correspond to four main themes. Chapter 1 (slides 2–7) documents the period before the accident in 2007; Chapter 2 (slides 8–20) includes the hospital and rehabilitation period immediately after the accident, as well as learning to cope and find support on a personal and physical level; Chapter 3 (slides 21–34) commences at the start of 2008 when the author returned to work as a teacher in a Special School; and finally, in Chapter 4 (slides 35–54), the author moves out of the safety of the community of people with disabilities to pursue the training of teachers as inclusive educators. It concludes with a review of the author’s perspective on life and thoughts on the future.

Study A ratified the author’s impression that learners were able to communicate with her on a more personal level, thus improving her effectiveness as a teacher of learners with disabilities, not through different teaching methodology but ‘rather that they saw a *connection* and felt more comfortable to talk with me’ (slides 27–34). Of the seven participants, five responded that there was an improvement in the author’s teaching, with two indicating that her teaching remained the same. It was noted that the two participants who did not feel that there was an improvement in teaching ability, still commented that the author exhibited sensitivity to participant’s needs. All of those indicating an improvement cited understanding and disability support as areas where the author was more sensitive and that the author was able to communicate on many levels with the learners as a result of experiencing disability.

In Study B, answers to Question 1 (Addendum A) revealed that 95 of the 115 students who completed the questionnaire felt that the author’s disability had a positive effect on lecturing Inclusive Education (slide 44), 19 felt that it had no effect and 1 participant was unsure. In response to Question 2 (Addendum A), 68 participants indicated that interaction with the author nurtured a positive view of people with disabilities, 46 indicated no effect, and again 1 was unsure (slide 45). When asked to evaluate whether the author having a disability and lecturing participants in Inclusive Education affected their teaching practice in any way (Question 3, Addendum A), 60 responded that there had been a positive effect, 52 felt there had been no change and 3 were unsure (slide 46).

## Ethical considerations

As the main participant in this study, the author draws on her experiences and analysis thereof, and chooses the elements of the story to be told (Hiles, Čermák & Chrz 2009); therefore, all views expressed are the author’s own, except where stated otherwise.

In order to complete Study A, ex-learners were approached via www.facebook.com. Only those 2010 Grade 12 learners, who had initiated friendship with the author through this medium after she was no longer their teacher, were included. All participants were over the age of 18. Again, the purpose of the study was explained and participants were invited to participate in Study A on a voluntary basis.

Full time tertiary student questionnaires were completed on a voluntary, anonymous basis. Prior to their completion of the questionnaires, the purpose of Study B was discussed and it was stressed that choice of participation in the study, or not, would not affect a student’s marks.

## Trustworthiness

The author’s bias implicit in the nature of narratives is acknowledged. Within the constructivist framework, each person perceives and constructs reality from a different viewpoint and thus this study shows only the author’s interpretation of reality within the South African context (Henning 2004). Study A and Study B were carried out, therefore, to assess the reliability of the author’s impressions of the impact of her disability on others in educational contexts.

## Discussion

### Chapter 1: Start from the very beginning

Here follows a little background to set the scene.

We all have talents, and mine seems to be in the area of communication. Right from the start, children with disabilities have always gravitated towards me. In my first year of teaching in 1998, I was blessed by having two children with special needs in my mainstream classroom, one with a cognitive impairment and the other with autism. I had never heard the words ’Inclusive Education’ at that stage; however, I believe that if a child is in your class, you have the responsibility to teach him or her.

After working as a private tutor with children on the Autistic spectrum, I decided to pursue my studies in teaching to complete my degree in education. With my new degree, I went to work at the Open Air School for learners with Physical Disabilities and continued my postgraduate studies part-time. During evening lectures, which often ended in debate, I was introduced to the Education White Paper 6, and I realised that teaching in a special school contradicted my belief in Inclusive Education, but I needed the experience, and aspire to the philosophy of trying to make a difference in whatever situation I may be.

As an able-bodied person within the special school context, the misperception of people that I had enormous amounts of patience to work with special needs children frustrated me. Rather than patience, I maintain firm boundaries and high expectations. In addition, I learned that ’children are children‘, no matter how they look from the outside.

Fiercely independent, passionate and driven, I usually achieved what I set out to do. With a passion for social justice, I generally enjoyed fighting the good fight, and challenging the social norms within both school and university spheres. At least that was how I saw myself.

### Chapter 2: Fragility

In 2007, I was involved in a car accident.

It was at that point that my world was turned upside down. In a single moment I became fragile; I became completely dependent on others, unsure of myself, needing a fixed routine to survive the day, and too afraid to go anywhere on my own.

My 3-month stay in hospital was excruciating. Separated from my family, unable to move and living on pain medication and antidepressants, I hit the lowest point of my life. I am quite sure that I am not the only one who has been in the condition where, if I could have actually moved myself out of the hospital bed, I would have thrown myself out of the window. And then, one night when breathing did not happen naturally, I realised that I wanted to live and forced myself to breathe … the wonders of bilateral pulmonary emboli.

I did most of my grieving in hospital and I came to terms with the fact that I would no longer be able to do all the things that I had previously been able to do; however, this has not always been possible for the members of my family. My now exhusband and my father both believed that I had given up hope, but in my eyes I had accepted the facts, which was important for me in order to be able to move forward.

Through the faith that I had in the children that I worked with, I learned to do some of the most difficult things I have ever had to do. I learned to self-catheterise, forced myself to climb into my wheelchair when my head spun and stood on burning feet, even if only for a moment.

Working in a school for children with disabilities, the disability I was now confronted with was neither foreign nor scary. That said, I daily make a conscious decision that I will live my life and not allow others to prevent me from achieving my dreams.

It was still difficult for me during that time to go anywhere in public where children stared and adults looked away from me.

I am deeply appreciative of the support systems I have. Without them, I would have had either no reason to live or perhaps no ability to live. At the innermost core exists my family, followed by my helpers and friends (commonly known as my ’friendamily‘); together, we are learning to multiply by three. Whilst my conscious mind understands that I cannot do everything that I did before, my unconscious does not. I have come to realise that it takes me now three times longer to do things than it did before. Finally, I have the professionals who have offered great support. These are the people in my life who catch me when the wheels fall off, and do not tell me what I cannot do, but just help me to succeed.

Unfortunately, not all aspects of my life were supportive or positive. My disability changed my relationships; for instance, my exhusband could not cope and used to throw my wheelchair around, and forced me to walk with crutches the entire length of a shopping centre so that I was unable to walk at all for days afterwards.

Just short of one year after the accident, I fell pregnant with my son Darrion. The frustrations that I experienced during this pregnancy were something I had no idea existed. I had to stop all of my medication, and my body reacted badly and eventually I lost 4 kg during my first 6 weeks of pregnancy. I was in permanent pain, which increased as the baby grew, and I could not take any pain medication. I spent my entire pregnancy in my wheelchair and developed asthma and the early stages of diabetes. Darrion was delivered 5 weeks early through an emergency caesarean, and weighed 3.6 kg. After a week, we went home. Two days later, my husband left.

Circumstances became progressively more difficult because nobody could tell me ways of coping with a new and growing baby from a wheelchair. For once, I found the Internet to be completely useless. And, once again, I thank the Lord for my family who stepped in and devoted time and energy to help me to work things out.

My expectations of myself as a mother are frequently still more than I am able to achieve. Even though I understand that I have limits, I have to rely on my family and friends to help me to carry out normal family activities. But I am determined that my children will not miss out because I am a single parent with a disability … ever the over-achiever.

Shopping is perhaps one of the most difficult tasks for me. Most of the malls in Durban are not as wheelchair-friendly as one would hope, although there has been a definite improvement in the allocation of parking bays. We sometimes spend 20 minutes waiting for a lift because every time it passes it is full, or in some places there is no lift and the wheelchair ramp is in the parking-lot which is really unpleasant on a rainy day.

In busy shops, I have often resorted to humour when I could not move through an aisle and I have to ask people to move their trolleys. I tend to make jokes about the size of my butt; this generally elicits a look of shock at first, and thereafter a smile. People in South Africa frequently still presume that if one is in a wheelchair one has a cognitive impairment.

One of my biggest frustrations, to this day, is the assumptions that people, who do not need wheelchairs, make about wheelchairs. They presuppose that wheelchair users are unhappy to be forced to use a wheelchair; however, for the person in a wheelchair that vehicle is often seen as a means to freedom.

It was only this year that I finally acquired a power wheelchair. Despite criticism, I love the difference it is making to my life.

I become tired of defending myself, my actions and my decisions. It is wearying to deal with the ’Ag, shame‘-mentality that still pervades the general South African population. I am frequently tempted to come back with a snappy retort, but it probably would not help! By attempting to break down barriers this way, well-meaning people would rather become defensive and it would more likely increase negative perceptions, rather than foster friendship, respect and understanding. Hours of therapy have helped me to understand that interacting with me sometimes brings up other people’s feelings of discomfort, and forces them to deal with the uncertainties of life.

### Chapter 3: One of us … the culture of people with disabilities in South Africa, our apartheid legacy

’Welcome back, Miss, you’re one of us now‘, was the joyful statement that greeted me on my return to the school where I taught, and that was how it felt. I found that the only place where I was *accepted* for exactly who I am and appreciated for what I could do, was the special school for children with physical disabilities where I worked. The learners form an isolated community of people with disabilities that has little interaction with the local community in which it exists, reminiscent of pre–1994 segregation. In retrospect, I was not really ready to return to work but I feared that I would turn into a recluse if I remained completely cut-off from society any longer. Leaving the house is a conscious decision that I still make on a daily basis.

No longer able to work with the younger children, where the physical demands were too strenuous, I was moved into teaching Mathematics at senior and high school level, and later Computer Applications Technology up to Grade 12.

I started to play the role of mentor to many of the teenagers, who turned to me with personal problems. They now felt that, with my experiences, I could understand what they were going through. This was most frequently noticeable amongst the girls who often remain silent in the patriarchal isiZulu communities which still exist in rural KwaZulu Natal.

During this time, I was also lucky enough to attend the SAALED mini-conference in Durban, where I discovered the work of Toni Noble and Helen McGrath (2003) in the development of *resilience*. I came to see this as one of the greatest areas of need amongst children with disabilities.

When considering the development of children born with disabilities in terms of Erikson’s psychosocial developmental stage of Autonomy versus Shame and Doubt (De Witt 2009), it seems to me that many of the learners that I taught had not developed the belief in their ability to perform tasks independently, which results in giving up at the first sign of possible failure.

I had witnessed this in pre-school to Grade 12 learners. It could be a consequence of overcompensation by parents and caregivers in this developmental stage, where they did for their children what those children could have accomplished for themselves. In so doing, they did not give their children the opportunity to develop the confidence to keep on trying until they experience success.

As a result, I staged a school-wide campaign based on the *Bounce-Back* programme to encourage learners not to accept defeat so easily and to accept that sometimes things will not go well, but that does not mean it is a catastrophe. (McGrath & Noble 2003; Dr Seuss 1990).

I also started a motivational wall where I placed quotes and inspirational sayings with a distinctly feminist flavour on a weekly basis. This especially appealed to the girls that I taught, although many times, I snuck up on the boys having a quick ‘squizz’ (look).

Finally, I started a movie club, where the goal was not to watch the latest releases, but to watch movies with social comment so that the learners could see themselves within the larger societal and world context. These Friday night movie evenings were restricted to those learners who stayed at the hostel over weekends, and included popcorn and a general discussion afterwards. This gave them an opportunity to express their views and to enter into discussion on how society had, and could still, change.

My focus here was to empower learners to accept themselves and to take ownership of the disability and responsibility for their learning. I modelled pro-active mantras such as ‘Disability is just a part of me, not my defining factor’, and taught learners to be assertive in expressing their particular needs and preferences with regard to accommodations needed in the classroom.

I was privileged to have had the opportunity to work with teenagers with disabilities in helping to identify limits, push the boundaries, and find alternate routes around obstacles. I introduced presentation software for delivering orals for learners who experience difficulties with verbal communication. I firmly embrace the Open Air school motto *‘I can and I will’* (Bishop cited in Morris 2011).

In order to ascertain whether my disability had any effect on my ability to teach learners with disabilities, I conducted a mini-study through facebook. My exlearners responded in mixed ways. Some felt that, because I was (and am) a good teacher, my disability did not improve my ability to teach. Additionally, most of the learners felt that I had a better understanding of the difficulties they experienced and that they could relate to me on a personal level and receive better classroom support. (Possibly because of my disability, they may have felt more comfortable speaking to me.)

As part of my learning process, I realised that I had greater limitations in terms of energy reserves than I had initially thought. My home life was suffering.

Passionate as I am about what I was doing, in the back of my mind, I still felt I could only help a limited number of children experiencing barriers to learning within the confines of the special school. I realised that I would need to leave this safe-haven for the bigger world where I could reach teachers and, through them, help exponentially more children. Ironically, the very identity which the learners bestowed upon me was the exact reason that I had to move on: being included by the learners in their community of people with disabilities, from which I had been excluded as an able-bodied teacher, motivated me to move out into a position where I could actively promote Inclusive Education, in the hope of helping to prevent other children to leave their home community to join a community of people with disabilities.

The next stage in my journey took greater will-power and stubborn determination than any thus far. I left the safety of my job and comfort zone to follow the same dream that I had set out for years before … but, as expected, took three times longer than anticipated. I had misgivings about this move, and frequently fear threatened to overwhelm me. I focused nevertheless on the thought that, if I could influence 60 children by myself in a special school, just imagine how many I could reach if I could reach teachers.

### Chapter 4: Rolling on with life

So it was that in July 2010, I moved to the Embury Institute for Teacher Education, a private university that specialises in teacher training. I had no idea of the physical strain it would exert on my body when I left a wheelchair-friendly environment to tackle stairs every day. Even now, I still have to make a conscious decision to leave my house every day. Unfortunately, my ability to reach others and make changes comes at a price, and I am not willing to wait for the changes to be made to welcome me in; by then I will have missed the boat.

Here, I have been supported in further developing a 2-year course on Inclusive Education, which forms part of the requirements for the Bachelor Degree in Foundation Phase Education. The modules are completed in the second and third years of study and focus on barriers to learning, including disabilities, from a practical perspective placed in a South African as well as in a global context from a human rights based perspective.

I lecture to two distinct groups of students:

- Full-time, undergraduate students. Generally these students come from urban areas and are, for the most part, English-speaking.
- Approximately 170 Grade R teachers from rural communities who are employed by the Department of Basic Education. These students attend lectures during school holidays and for 1 week per term, and are, for the most part, isiZulu-speaking.

These students form the sample group for the later mini-research project carried out by myself to determine whether my disability influences my effectiveness in the lecture room.

In my experience there is a lack of exposure to people with disabilities playing assertive roles in our everyday communities. It is most likely because of historical segregation and discrimination, that when people are exposed to disability, they react with heightened sensitivity, discomfort and sympathy. Instead of offering assistance in a respectful way, they are either overbearing or too uncomfortable to act in a logical manner. Frequently, people will intentionally look away or over the head of someone with a disability, or respond with an ’Ag, shame‘.

This ’Ag, shame‘-mentality pervades South African society and has to be one of my most frustrating experiences on both a personal and a professional level. I am most appreciative to those people who respond in a level-headed, logical manner when faced with someone with a disability.

It has been my goal to desensitise students to disability, by drawing attention to and from my disability, yet raise awareness of social justice by teaching them how to teach in a respectful, professional, and empathetic manner.

At an academic level, my objective here was to help teachers see that inclusion in South Africa is not only doable, but good for learners, and best practice for teachers. This will ultimately direct classroom practices towards both inclusivity and quality.

The modules that I have curriculated draw on four main sources:

- Firstly, the South African Department of Basic Education establishes the policy, perspective and protocol (South African Department of Basic Education 1995, 2001, 2008, 2009).
- UNESCO Bangkok (2009) provides an international view on ‘Teaching children with disabilities in inclusive settings’, providing clear and accessible information for second language English-speakers.
- Bornman and Rose (2010) bring home the South African perspective on the practicalities of including learners experiencing barriers to learning.
- Fourthly, my personal classroom and life experiences colour our discussions with real problems and solutions and my students are free to explore my failures and successes to realise that, although not everything we do will be successful, there are many more things to try.

My aim within these readings and lectures is to empower teachers to draw on *practical creativity* to source, research and create solutions to barriers to learning within their classrooms, school, district and community.

As a means of encouraging interaction with the texts and encouraging critical thinking and discourse, students are required to write reflections on the required readings and to participate in discussions. As a result, many of the students have shared their experiences, bringing the reality of the classroom to the academic environment of the lecture theatre.

Within the comfort of the lecture room, we directly tackle some of the myths regarding disabilities prevalent in South African society, such as albinism, HIV and disability, through discussions where we explore housewives-tales and other myths. Students are empowered with knowledge and facts that they can disseminate to their schools and communities.

The final role that I have found myself playing is that of counsellor. My knowledge of inclusive strategies, as well as my general approachability, encourages students to seek assistance and support and there is often a queue outside my office. *That* is what Inclusive Education is really about: co-operatively supporting each other.

In order to gauge the impact of my disability on my effectiveness in lecturing on Inclusive Education, I conducted a small study on my students through a voluntary, anonymous semi-open-ended questionnaire (Addendum A).

The results of the study were highly gratifying:

- Of the 115 students who responded, 83% felt that my disability impacted positively on the effectiveness of my lecturing on Inclusive Education. One of the themes evident in students’ comments was that my disability gave them better insight into the daily barriers experienced by learners. This reiterates the belief that people with disabilities need to be in the forefront of disability and Inclusive Education.
- 59% of students felt that my disability and lecturing on Inclusive Education was responsible for changing their attitude towards persons with disabilities. Much to my relief, many of those who indicated that my having a disability had nothing to do with changing their attitude commented that they already had a positive attitude towards working with children with disabilities. This led me to conclude that inclusion training has a positive effect on the attitudes of teachers and future teachers in KwaZulu Natal.
- Finally, in the area of change in teaching practices, 52% said that there had been a change in their methodology of teaching. If one out of every two teachers changes his or her teaching practices after exposure to people with disabilities and Inclusive Education training, the pace at which classrooms become inclusive would be rapidly and positively affected by Inclusive Education training. This leaves opportunity for further study into the effectiveness of change on teaching practices where on-going support is provided for educators.

On a personal level, I have found wearing this hat to be most rewarding and fulfilling. Private sector work inevitably includes its own stress factors, with deadlines to meet and not enough seconds in each minute to complete all I wish to do; however, it also offers the opportunity to be on the cutting edge and the freedom to spread my wings.

The year 2011 has been one of growth:

- I have flown to other parts of our country twice already to attend conferences on Inclusive Education and it is wonderful to see that the seed is finally starting to sprout.
- As part of a group of passionate SAALED members, we recently formed a South African Association for Learning and Educational Difference (SAALED) KZN branch, where I have taken up the post of deputy chair. Our aims are to share information regarding best practice in terms of Inclusive Education, as well as to embark on outreaching into rural schools to provide support to teachers who are unable to attend training workshops in major city centres. I look forward to the fruit it will bear as inclusive practice becomes more rooted in classrooms across our beloved country.

## Conclusion

### Drawing the curtain

Whilst writing this conclusion, I happened across a TED talk by Stuart Brown (2009) on the role of playing, and made a wonderfully revealing discovery about myself: I love to play.

One of the directors asked me last year, in all seriousness, what I was doing … to which I, in all seriousness, responded, ‘playing nicely’. Of course, I then had to support the statement with ‘If my work becomes a job, I won’t enjoy it anymore’. By viewing work as a form of play, it allows me to be more creative in what I do, rather than needing the rigid structure I needed in the first few years after my accident.

I have always loved toys; that was probably why I became a pre-school teacher to start with! I adore anything with a remote control. I love building with Lego and changing transformers, but now my toys have changed and I am starting to enjoy my life more. I can take a walk to the shop (power chair); I can talk to my computer (using Dragon software); I can develop new and interesting courses. The students love them because they are fun, problem-focused and interesting. I can still read to my kids. And like Thomas the Tank Engine, I serve a purpose.

Perhaps this is one of the reasons that I have been able to adapt, because I have not lost my sense of fun. I love to solve problems: word problems, math problems, learning problems and teaching problems. My work, to me, is serious fun! It is one of the reasons I am effective at what I do. It is probably the reason I manage to change people’s views, because to me ‘better’ means fun, learning should be fun and fun means ‘inclusion’, and teaching is all about having serious fun in the classroom.

Overcoming the daily obstacles is an ongoing process for all persons with disabilities, and many of these obstacles lie in the minds and perceptions of those around us. Sharing this journey is a blessing. I still live in pain every day of my life, and some days I rail against my dependence on others to live through the day; I grieve in my fragile humanity. On the other hand, each day I get out of bed, knowing that I do not have a choice … what I do makes a difference in someone’s life, and that I have to create a better future for others. Perhaps, because of something that I have said or affected, one child less will leave their family and their community, and one more child will grow up as part of a group of friends with hopes and dreams just like everyone else.

It should not be an anomaly when a person with a disability is successful. Ideally, it should be the standard expectation for all people to have the potential to achieve. It is generally accepted that teacher expectation of learner potential and the teacher’s belief in their ability to teach the learner directly impacts on learner achievement; therefore the focus needs to be placed on pro-actively equipping teachers to implement Inclusive Education so that teachers can believe that they have the ability to teach learners with disabilities and that those learners will achieve something.

In my opinion, equity in South Africa still has a long way to go but in the infancy of our democracy, baby steps have already been made:

- We have a constitution that acknowledges the rights of all policy, in terms of White Papers, the SIAS documents and Guidelines for Inclusive or Full Service Schools, have been put in place (DBE, 1995, 2001, 2008, 2009).
- Teacher training and tertiary institutions are fostering understanding.
- Associations such as SAALED, are supporting teachers to adopt best practice, that is, practices which have been proven to be effective, in their classrooms.

As a pro-active participant in developing an Inclusive Education system, it is my view that this train has left the station, and if the Department of Education continues to forge ahead, there is a good chance that in the very near future, our schools will be inclusive. It is, however, up to individuals to ensure that teachers are on that train, not just watching it disappear into the distance. As an individual with a disability, I feel that my choices to move out of my safety zone towards attaining Inclusive Education are worth the struggle, and I intend to trudge on.

Whilst much of my essence has been swept away, a few traits have remained: my dry, quirky sense of humour, my stubborn determination, and my demand for a better future. I do not want to rid myself of my disability; I want to change the world around me to suit me, because I am not the only one like me.

## Addendum A

Dear Student

I am trying to see if my disability makes me more effective as a lecturer in the area of Inclusive Education and it would be most helpful to me if you would fill in this questionnaire. This is a voluntary, anonymous questionnaire which will have no effect on your marks whatsoever. If you do not wish to fill it in, simply return the blank form with all the others. Your participation will be enormously appreciated.

Thank you

Helen Laas

__________________________________________________

### Question 1

Do you think that my having a disability has had any effect on the effectiveness of my lecturing in Inclusive Education? That is, do you think I am a better lecturer in Inclusive Education because I have a disability? (Please tick)

Yes No

Comment:

__________________________________________________

__________________________________________________

__________________________________________________

__________________________________________________

__________________________________________________

__________________________________________________

__________________________________________________

__________________________________________________

__________________________________________________

### Question 2

Has my having a disability affected your attitude and feelings towards people with disabilities?

Yes No

If yes, please could you tell me how?

__________________________________________________

__________________________________________________

__________________________________________________

__________________________________________________

__________________________________________________

__________________________________________________

__________________________________________________

### Question 3:

Has my having a disability and lecturing you in Inclusive Education affected your teaching practice in any way?

Yes No

If yes, could you please share an example of how?

__________________________________________________

__________________________________________________

__________________________________________________

__________________________________________________

### Any other comments:

__________________________________________________

__________________________________________________

__________________________________________________

__________________________________________________

__________________________________________________

__________________________________________________
